# Biochemical and Plasma Lipid Responses to Pemafibrate in Patients With Primary Biliary Cholangitis and Dyslipidemia: A Four-Year Analysis

**DOI:** 10.7759/cureus.83176

**Published:** 2025-04-29

**Authors:** Hiroyuki Kobayashi, Ayumi Sugiura, Mizuki Koyama-Otagiri, Takayuki Nimura, Yukifumi Kurasawa, Haruaki Shirakawa, Satoru Joshita

**Affiliations:** 1 Gastroenterology, Shinshu University School of Medicine, Matsumoto, JPN; 2 Internal Medicine, Sato Hospital, Nakano, JPN; 3 Internal Medicine, NHI Yodakubo Hospital, Nagawa, JPN

**Keywords:** bezafibrate, dyslipidemia, pemafibrate, primary biliary cholangitis, ursodeoxycholic acid

## Abstract

Background and aims

Primary biliary cholangitis (PBC) is a chronic autoimmune liver disease treated with ursodeoxycholic acid (UDCA), though some patients respond inadequately. This study evaluated the mid-term effects of pemafibrate on liver function and lipid profiles in PBC patients with dyslipidemia who were refractory to UDCA alone or with bezafibrate.

Methods

A retrospective review was conducted on 25 PBC patients (17 female; median age: 71) who began treatment with pemafibrate and UDCA at Shinshu University Hospital or NHI Yodakubo Hospital in 2021. Patients were either given pemafibrate as an add-on to UDCA (n = 10) or switched from UDCA + bezafibrate (n = 15). Biochemical markers were monitored over four years.

Results

Median alkaline phosphatase (ALP) declined from 138 U/L to 85, 78, 82, and 77 U/L at years 1-4, respectively. ALP normalization increased from 36% to 86% over the same period (P < 0.001). Gamma-glutamyl transferase dropped from 53 to 36 U/L at one year and remained stable. Alanine aminotransferase improved similarly (26-19 U/L, P = 0.007). No significant changes were seen in aspartate aminotransferase, bilirubin, creatinine, or estimated glomerular filtration rate (eGFR). No serious adverse effects were reported.

Conclusions

Pemafibrate with UDCA led to sustained liver enzyme improvement and was well-tolerated in dyslipidemic PBC patients refractory to standard therapy. Prospective studies are warranted to evaluate its long-term benefits, including in patients without dyslipidemia.

## Introduction

Primary biliary cholangitis (PBC) is a chronic liver disease caused by yet undetermined autoimmune mechanisms. PBC primarily afflicts middle-aged and older women and is characterized by the presence of disease-specific anti-mitochondrial antibodies (AMAs) [1]. Genetic and environmental influences along with epigenetic alterations are suspected as disease susceptibility factors [2-4]. PBC displays chronic destruction and inflammation of the interlobular bile ducts, leading to cholestasis and eventual progression to cirrhosis, liver failure, and, in rare cases, hepatocellular carcinoma (HCC) [5-8].

The vast majority of PBC patients are asymptomatic at the time of diagnosis and show a favorable response to ursodeoxycholic acid (UDCA), which is currently a first-line treatment for PBC and recommended by many guidelines [9-11] for liver biochemistry improvement and slower histological progression to liver cirrhosis [12,13]. However, some patients exhibit an inadequate therapeutic response to UDCA in the clinical setting. Bezafibrate addition to UDCA therapy has been demonstrated to achieve a favorable biochemical response and better prognosis in refractory PBC [14-16]. This regimen has been recommended for UDCA-unresponsive patients by the Japan Society of Hepatology (JSH) guidelines despite not being approved by Japanese national health insurance [9]. However, bezafibrate causes adverse hepatic as well as neuromuscular and skeletal reactions, along with increased serum aspartate aminotransferase (AST) and creatine phosphokinase (CPK) levels, in over 10% of patients.

As a new selective peroxisome proliferator-activated receptor alpha modulator, pemafibrate has very recently been approved for the treatment of dyslipidemia [17,18]. This agent is primarily prescribed for hypertriglyceridemia and low high-density lipoprotein cholesterol (HDL-C) to improve lipid profiles in the blood. Compared with conventional fibrates, pemafibrate offers higher safety and fewer side effects, while being well tolerated by patients with impaired renal function. To date, four reports including our own have described the short-term clinical results of pemafibrate addition therapy on liver function in PBC patients [19-22]. However, the mid-term effects of pemafibrate on PBC with accompanying dyslipidemia have not yet been examined.

The present study investigated the four-year biochemical and plasma lipid responses of pemafibrate in PBC patients with dyslipidemia who showed an incomplete response to UDCA alone or in combination with bezafibrate.

## Materials and methods

Patients targeted for analysis

**Figure 1:**
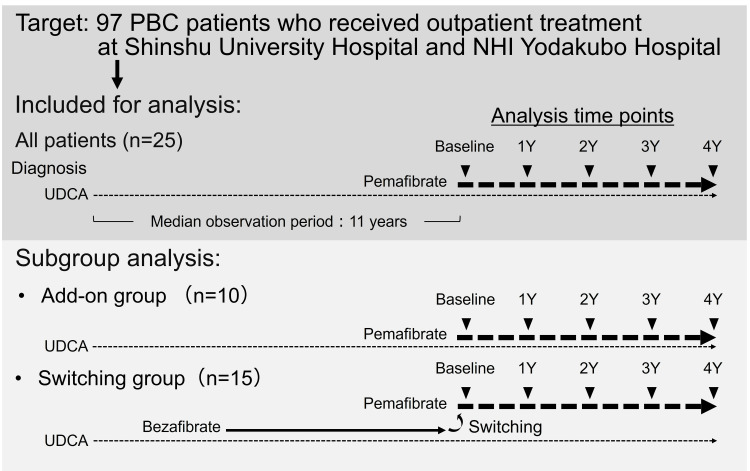
Study participant selection. PBC: primary biliary cholangitis; UDCA: ursodeoxycholic acid

We conducted a retrospective chart review of 97 patients with PBC (81 female, median age at the 2021 calendar year: 71 years, median age at diagnosis: 56 years) who were receiving medical care at Shinshu University Hospital or NHI Yodakubo Hospital in 2021. All patients had been diagnosed as having PBC based on criteria established by the JSH [9]. No patient had a history of organ transplantation or the concurrent use of immunomodulatory drugs or corticosteroids, and none were coinfected with the hepatitis C or B virus or exhibited evidence of alcoholic liver disease or non-alcoholic fatty liver disease. Among them, 25 patients (17 female, median age at the 2021 calendar year: 71 years, median age at diagnosis: 60 years) had already begun combination therapy of UDCA and pemafibrate in 2021, all of whom were indicated for pemafibrate treatment of dyslipidemia according to their attending physician (Figure 1, Table 1). The median observation period between the diagnosis of PBC and the start of pemafibrate was 11 years. Ten patients had already been treated with UDCA alone and were prescribed pemafibrate in addition to UDCA (add-on group), while 15 patients had already been treated with UDCA and bezafibrate and were switched to UDCA and pemafibrate (switching group). Oral 0.1 mg pemafibrate was administered to all patients twice daily.

**Table 1:** Patient characteristics (categorical variables) and comparisons between the time point of PBC diagnosis and baseline in 2021. PBC: primary biliary cholangitis; UDCA: ursodeoxycholic acid

	Time point of PBC diagnosis	Baseline in 2021
Female n (%)	17 (68%)	
Maintenance therapy for PBC		
UDCA 400 mg/600 mg/900 mg n (%)		1 (4%)/8 (32%)/16 (64%)
Bezafibrate 400 mg n (%)		15 (60%)

Reference range of alkaline phosphatase (ALP) and definition of incomplete ALP response

All serological testing was performed using standard methods. Since ALP measurements were obtained during the transition period from the Japanese Committee for Clinical Laboratory Standards (JFCC) to the International Federation of Clinical Chemistry and Laboratory Medicine (IFCC), ALP values measured by the JFCC were converted to IFCC values by multiplication by 0.35. According to the IFCC reference values, ALP > 113 U/L was defined as an incomplete response to medical therapy, with ALP ≤ 113 defined as ALP normalization.

Indications for pemafibrate add-on or switching

According to chart review, pemafibrate was initiated by the attending physician for the following strategies: (1) in patients with an incomplete response to UDCA alone as well as an indication for pemafibrate treatment of dyslipidemia, pemafibrate was added onto UDCA therapy (add-on group), and (2) in patients with an incomplete response to UDCA plus bezafibrate combination therapy, a switch to pemafibrate on UDCA therapy (switching group) was made in reference to a report describing adverse events for bezafibrate, which included increased creatinine kinase (CK) levels and myalgia [14].

Study ethics

The protocol of this study was reviewed and approved by the Institutional Review Board of Shinshu University School of Medicine (no. 6409) and conducted according to the principles of the Declaration of Helsinki (revised in 2013 by Fortaleza) and the Ethical Guidelines for Medical Research Involving Human Subjects (partially revised on February 28, 2017). An opt-out system is in place at our institution. All information on the protocol and conduct of the study, including its purpose, is available on the Department of Medicine, Shinshu University School of Medicine website (http://www.shinshu-u.ac.jp/faculty/medicine/chair/i-2nai/) as well as on the NHI Yodakubo Hospital website (https://www.yodakubo-hp.jp/). Patients not wishing to participate in the research were freely able to opt out of the study.

Statistical analysis

Statistical analysis and data visualization were carried out using StatFlex ver. 7.0.11 software (Artech Co., Ltd., Osaka, Japan). Continuous data at the time point of PBC diagnosis and at pemafibrate initiation (baseline) are expressed as the median and interquartile range. The Wilcoxon signed-rank test was employed to analyze the differences among continuous variables between the time points of PBC diagnosis and baseline. The changes in biochemical data after starting pemafibrate were analyzed for significance at baseline, one year (1Y), two years (2Y), three years (3Y), and four years (4Y) by means of the Friedman test. For items displaying significant differences, the Dunn-2 test was employed for post hoc analysis. The ALP normalization rate is analyzed using the chi-squared test. All statistical tests were two-sided and evaluated at the 0.05 level of significance. At 3Y, pemafibrate treatment was discontinued in one patient due to progression to liver cirrhosis. At 4Y, pemafibrate treatment was halted in four patients for reasons of progression to liver cirrhosis, HCC, death, and dropout, respectively. Clinical indices at these time points were calculated and compared among the remaining patients.

The fibrosis-4 (FIB-4) index and AST-to-platelet ratio index (APRI) were calculated as previously reported [23,24].

## Results

Clinical characteristics at PBC diagnosis

The clinical characteristics of the 25 patients at PBC diagnosis (17 female and eight male, median age: 60 years) are summarized in Tables 1, 2. Twenty-one patients (84%) were AMA-positive. The median ALP was 170 U/L, which was 1.51 times the upper limit of the reference range. Twenty patients (80%) had ALP levels exceeding the upper limit of the normal range. Thereafter, one, eight, and 16 patients were treated with 400, 600, or 900 mg/day UDCA, respectively, before pemafibrate addition. Bezafibrate had been added in 15 patients after PBC diagnosis but before baseline.

**Table 2:** Patient characteristics (continuous variables) and comparisons between the time point of PBC diagnosis and baseline in 2021. *The Wilcoxon signed-rank test was employed to analyze the differences between the time points of PBC diagnosis and baseline. **The time period from the time point of PBC diagnosis to baseline in 2021. PBC: primary biliary cholangitis; IQR: interquartile range; ALP: alkaline phosphatase; GGT: gamma-glutamyl transferase; ALT: alanine aminotransferase; AST: aspartate aminotransferase; eGFR: estimated glomerular filtration rate; TC: total cholesterol; HDL-C: high-density lipoprotein cholesterol; LDL-C: low-density lipoprotein cholesterol; TG: triglycerides; AMA: anti-mitochondrial antibody; FIB-4: fibrosis-4 index; APRI: AST-to-platelet ratio index

	Time point of PBC diagnosis		Baseline in 2021		
	Median	IQR	Median	IQR	P-value*
Age (years)	60	54-64	71	70-77	
Follow-up period (years)**			12	8-17	
Laboratory data					
Platelet count (x10^4^/µL)	22.7	16.5-26.7	17.8	13.8-24.9	0.017
Albumin (g/dL)	4.2	4.0-4.3	4.0	3.8-4.4	0.073
ALP (U/L)	170	123-23	138	76-182	0.031
GGT (U/L)	136	84-199	53	37-98	0.001
ALT (U/L)	42	29-69	26	17-39	<0.001
AST (U/L)	41	29-53	30	25-43	0.025
Total bilirubin (mg/dL)	0.76	0.52-1.04	0.68	0.58-0.89	0.357
Creatinine (mg/dL)	0.67	0.49-0.84	0.67	0.58-0.90	0.089
eGFR (mL/min/1.73 m²)	77	68-95	74	61-84	0.027
TC (mg/dL)	211	192-230	196	177-221	0.010
HDL-C (mg/dL)	56	42-67	63	48-73	0.271
LDL-C (mg/dL)	118	98-152	99	85-125	0.001
TG (mg/dL)	99	82-162	102	75-168	0.303
IgM (mg/dL)	322	158-572			
IgG (mg/dL)	1570	1,322-1,821			
FIB-4	1.64	1.17-2.51	2.59	1.70-3.89	0.010
APRI	0.49	0.29-0.72	0.41	0.29-0.74	0.392

Clinical characteristics at baseline

The median age was 71 years at the pemafibrate addition time point, with a median follow-up period of 12 years between the time point of PBC diagnosis and baseline in 2021 (Tables 1, 2). The median ALP level of 170 U/L had decreased significantly to 138 U/L at baseline (P = 0.034) (Tables 1, 2), which was 1.22 times the upper limit of the normal range, under UDCA therapy or UDCA with bezafibrate addition. The proportion of ALP abnormality was decreased, albeit not significantly (80% at diagnosis vs. 64% at baseline, P = 0.208), which indicated that the cohort receiving pemafibrate was a UDCA-refractory population. According to the Paris II criteria [25], 11 of 25 patients (44%) were defined as incomplete responders at baseline.

Biochemical changes after pemafibrate addition

**Figure 2:**
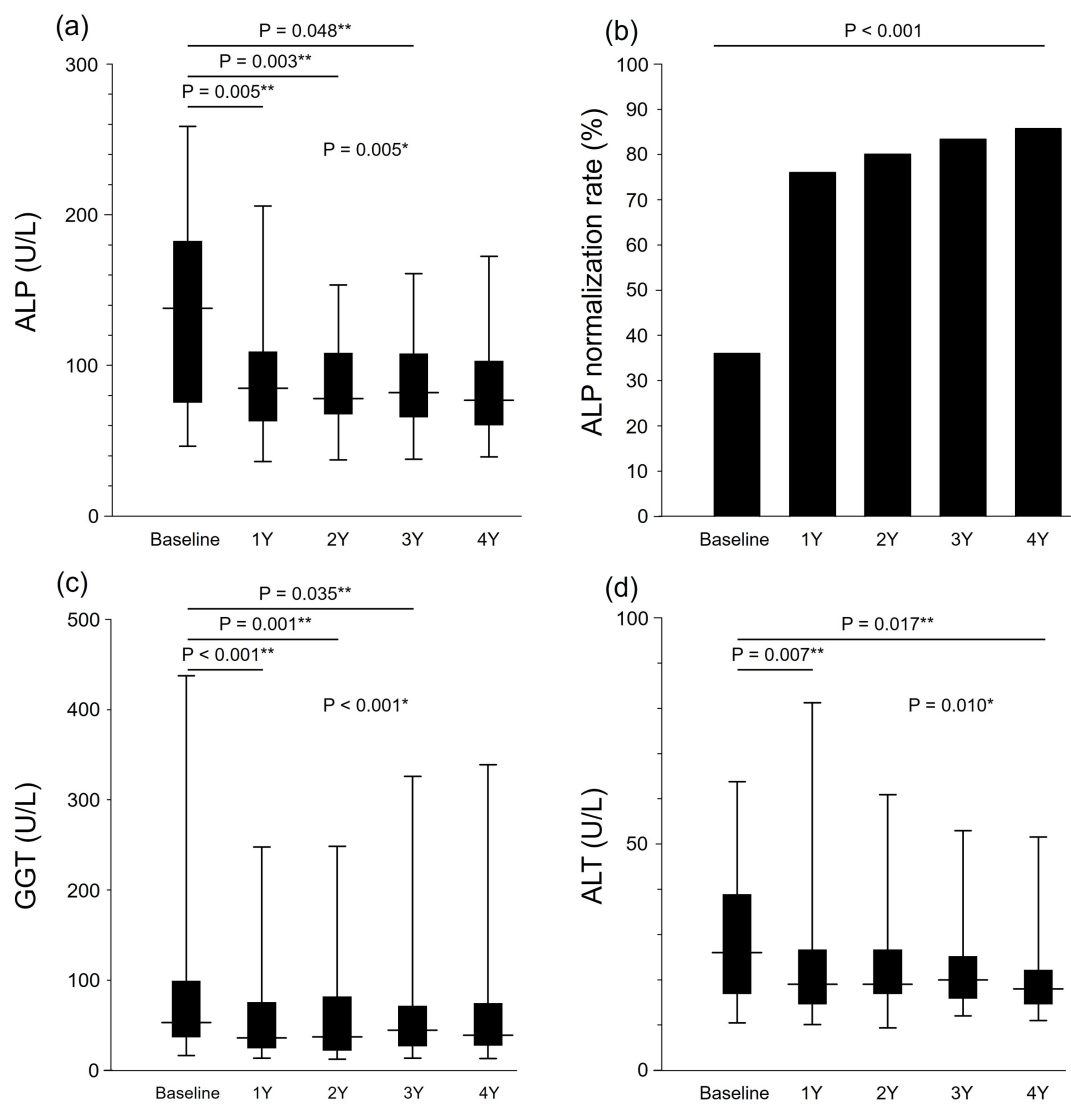
Yearly biochemical changes from baseline to 4 years for (a) ALP, (b) ALP normalization rate, (c) GGT, and (d) ALT (n = 25). *The changes in biochemical data after starting pemafibrate were analyzed for significance at baseline, 1 year (1Y), 2 years (2Y), 3 years (3Y), and 4 years (4Y) by means of the Friedman test. **The Dunn-2 test was employed for post hoc analysis. The ALP normalization rate is analyzed using the chi-squared test (b). ALP: alkaline phosphatase; GGT: gamma-glutamyl transferase; ALT: alanine aminotransferase

Median ALP levels decreased significantly from 138 U/L at baseline to 85 U/L at 1Y, 78 U/L at 2Y, 82 U/L at 3Y, and 77 U/L at 4Y (P = 0.005, Friedman test). ALP became significantly decreased at 1Y (P = 0.005), 2Y (P = 0.003), and 3Y (P = 0.048), but not at 4Y (P = 0.220), in Dunn-2 testing (Figure 2a). These improvements led to sustained ALP stabilization along with a steadily increasing ALP normalization rate from 36% at baseline to 76% at 1Y, 80% at 2Y, 83% at 3Y, and 86% at 4Y, which reached statistical significance (P < 0.001, chi-squared test) (Figure 2b).

Median gamma-glutamyl transferase (GGT) levels also decreased significantly from 53 U/L at baseline to 36 U/L at 1Y, 37 U/L at 2Y, 45 U/L at 3Y, and 39 U/L at 4Y (P < 0.001, Friedman test). GGT levels became significantly decreased from baseline at 1Y (P < 0.001), 2Y (P = 0.001), and 3Y (P = 0.035), but not at 4Y (P = 0.334), in Dunn-2 testing (Figure 2c).

Median alanine aminotransferase (ALT) levels decreased significantly from 26 U/L at baseline to 19, 19, 20, and 18 U/L at 1Y-4Y, respectively (P = 0.010, Friedman test). ALT levels became significantly decreased from baseline at 1Y (P = 0.007) and 4Y (P = 0.017), but not at 2Y (P = 0.230) or 3Y (P = 1.000), in Dunn-2 analysis (Figure 2d).

**Figure 3:**
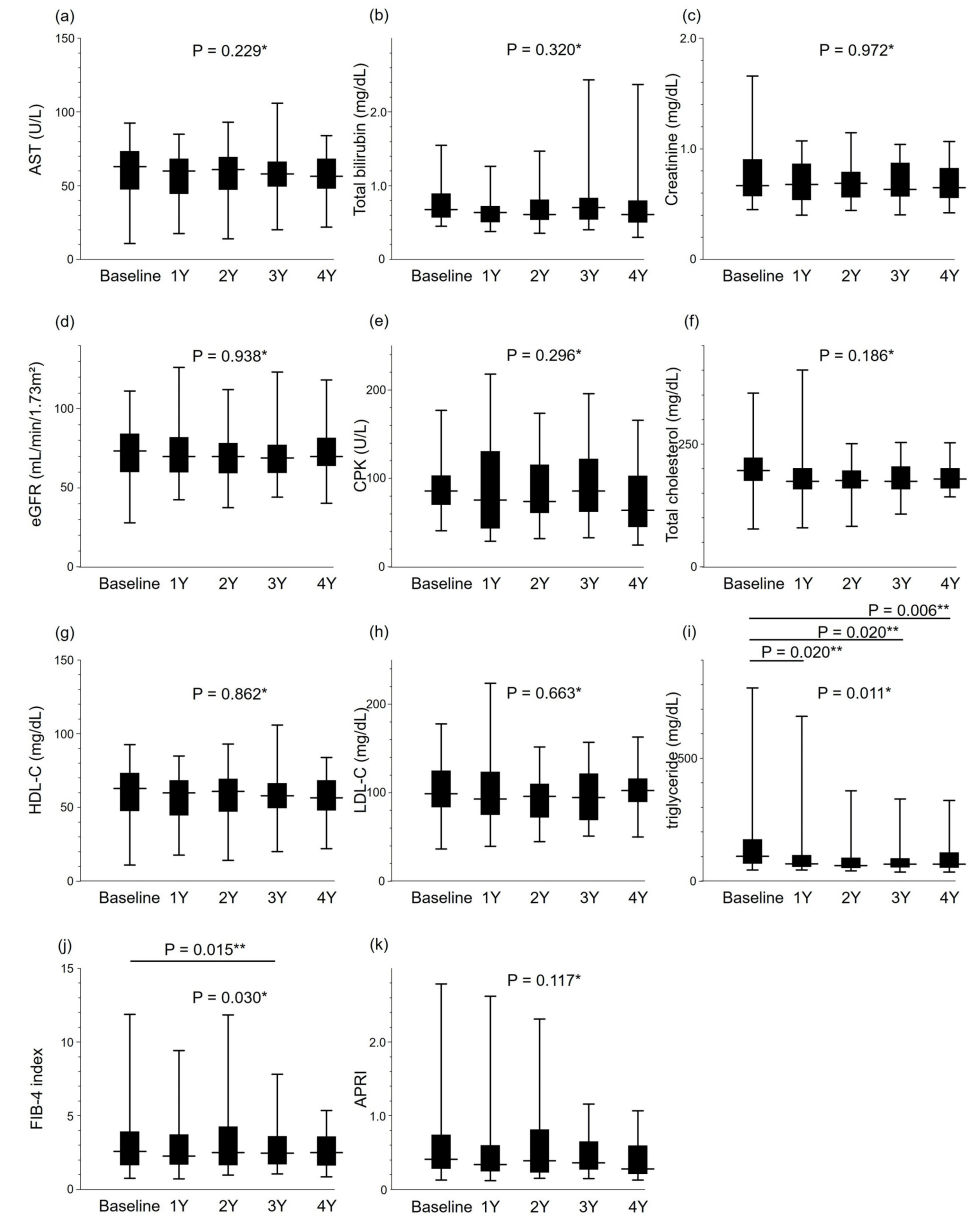
Yearly biochemical and plasma lipid changes from baseline to 4 years for (a) AST, (b) T-bil, (c) creatinine, (d) eGFR, (e) CPK, (f) total cholesterol, (g) HDL-C, (h) LDL-C, (i) triglycerides, (j) FIB-4, and (k) APRI (n = 25). *The changes in biochemical data after starting pemafibrate were analyzed for significance at baseline, 1 year (1Y), 2 years (2Y), 3 years (3Y), and 4 years (4Y) by means of the Friedman test. **For items displaying significant differences, the Dunn-2 test was employed for post hoc analysis. AST: aspartate aminotransferase; T-bil: total bilirubin; eGFR: estimated glomerular filtration rate; CPK: creatine phosphokinase; HDL-C: high-density lipoprotein cholesterol; LDL-C: low-density lipoprotein cholesterol; FIB-4: fibrosis-4 index; APRI: AST-to-platelet ratio index

We observed no significant changes in the levels of AST, total bilirubin, creatinine, estimated glomerular filtration rate (eGFR), or CPK in Friedman testing (Figures 3a-3e).

Lipid changes after pemafibrate addition

The addition of pemafibrate imparted no significant changes in total cholesterol (Figure 3f), HDL-C (Figure 3g), or low-density lipoprotein cholesterol (Figure 3h) but produced a significant change in triglycerides (TG) (Figure 3i) by the Friedman test. Median TG levels became significantly decreased from baseline at 102 mg/dL to 71 mg/dL at 1Y (P = 0.020), 64 mg/dL at 2Y, 70 mg/dL at 3Y (P = 0.020), and 69 mg/dL at 4Y (P = 0.006) in Dunn-2 testing (Figure 3i).

Fibrosis index changes after pemafibrate addition

Pemafibrate addition produced a significant change in the FIB-4 index (P = 0.030, Friedman test) (Figure 3j), but not in APRI (P = 0.117) (Figure 3k). Median FIB-4 levels were 2.59 at baseline, 2.28 at 1Y, 2.50 at 2Y, 2.47 at 3Y, and 2.51 at 4Y, with a significant difference between baseline and 3Y in Dunn-2 testing (P = 0.015) (Figure 3j).

Biochemical and plasma lipid changes in the add-on and switching groups

**Figure 4:**
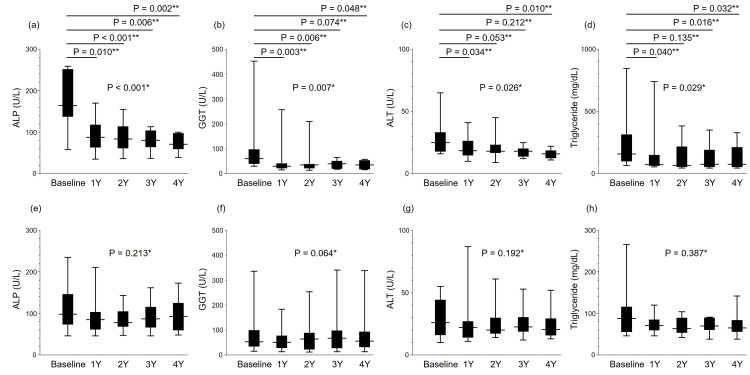
Yearly changes from baseline to 4 years in the pemafibrate add-on group for (a) ALP, (b) GGT, (c) ALT, and (d) triglycerides (n = 10) and in the switching group for (e) ALP, (f) GGT, (g) ALT, and (h) triglycerides (n = 15). *The changes in biochemical data after starting pemafibrate were analyzed for significance at baseline, 1 year (1Y), 2 years (2Y), 3 years (3Y), and 4 years (4Y) by means of the Friedman test. **For items displaying significant differences, the Dunn-2 test was employed for post hoc analysis. ALP: alkaline phosphatase; GGT: gamma-glutamyl transferase; ALT: alanine aminotransferase

Subgroup analysis of the add-on group demonstrated significant differences in ALP (P < 0.001), GGT (P = 0.007), ALT (P = 0.026), and TG (P = 0.029) by the Friedman test (Figures 4a-4d). In Dunn-2 testing, ALP levels at baseline became significantly decreased at 1Y (P = 0.010), 2Y (P < 0.001), 3Y (P = 0.006), and 4Y (P = 0.002). GGT levels at baseline also became significantly lower at 1Y (P = 0.003), 2Y (P = 0.006), and 4Y (P = 0.048). ALT levels at baseline became significantly decreased at 1Y (P = 0.034) and 4Y (P = 0.010). Baseline TG levels were significantly decreased at 1Y (P = 0.040), 3Y (P = 0.016), and 4Y (P = 0.032).

In the switching group, no significant differences were found in ALP, GGT, ALT, or TG (Figures 4e-4h), while a significant difference was found in FIB-4 only (P < 0.001) by the Friedman test, with no significance at any time point in Dunn-2 testing.

Adverse drug reactions after pemafibrate addition

No patient experienced myalgia or renal dysfunction after pemafibrate addition. Serum CK levels showed no significant increases after pemafibrate addition at any time point. Similarly, creatinine and eGFR showed no remarkable increases from baseline despite yearly patient aging. Based on the review of the medical records, there was no significant change in the pruritus.

## Discussion

This study analyzed the changes in biochemical test values and lipid markers over a four-year period during pemafibrate treatment in patients with PBC complicated with dyslipidemia. After pemafibrate initiation, ALP, GGT, and ALT all showed significant improvements, with the normalization rate of ALP gradually increasing over the treatment period.

The prognosis of patients with PBC has improved greatly in recent decades due to earlier diagnosis and the widespread use of UDCA therapy. As a result, patients exhibiting a favorable biochemical response to UDCA may even expect a normal life expectancy [26]. Among the 25 patients in this study cohort, 20 (80%) displayed abnormal ALP levels at the time of diagnosis. Most guidelines recommend UDCA as a first-line treatment in PBC for ALP normalization [9-11]. The guidelines from Europe and the US advocate 13-15 mg/kg/day UDCA, while those in Japan recommend 600 mg/day as an initial dose, without exception. Indeed, all patients in the present study received at least 600 mg/day of UDCA apart from one case of 400 mg/day. Of these, 15 patients had already received bezafibrate in combination with UDCA following an incomplete response to UDCA monotherapy. At the time of pemafibrate addition, ALP abnormalities had decreased in 16 out of 25 cases (64%), suggesting that earlier improvements were insufficient. Previous reports have demonstrated ALP amelioration in PBC patients who were refractory to UDCA monotherapy or UDCA and bezafibrate combination therapy within a short period after commencing pemafibrate [19-22]. The present study confirmed that ALP along with GGT significantly decreased after pemafibrate addition, thereby contributing to increased ALP normalization for at least four years. Additional long-term observational studies are needed to validate the clinical impact of combination therapy, including patient prognosis. In fact, a clinical trial evaluating the therapeutic effects of pemafibrate administration is currently underway [27].

PBC is a slowly progressive chronic liver disease with an etiology that remains incompletely understood and an absence of any established disease-modifying therapy. In our study, stepwise improvements in ALP levels were observed for at least four years during pemafibrate administration in UDCA-unresponsive PBC patients. Decreased ALP has been associated with improved prognosis in PBC, suggesting pemafibrate as a promising option for disease management. Although FIB-4 also showed a significant overall change, its differences at each time point were not statistically significant; further study is needed to evaluate the antifibrotic effects of pemafibrate. Since FIB-4 is influenced by age, its elevation in elderly patients does not necessarily indicate fibrosis progression. More accurate assessments of pemafibrate’s antifibrotic effects will require the addition of liver stiffness measurements, such as FibroScan and shear wave elastography [28,29], and other fibrosis markers [13,30].

Obeticholic acid in combination with UDCA has been approved in several countries as a treatment for patients displaying an inadequate response to UDCA [31] or drug intolerance [32]. As this option is unavailable in Japan, other alternatives are required to treat such cases. The JSH guidelines recommend the administration of bezafibrate, which is not approved for national health insurance coverage as a treatment for PBC alone. Therefore, in patients without concomitant dyslipidemia, bezafibrate use should be limited to clinical research. Recent studies showed that UDCA with bezafibrate could produce a complete biochemical response versus UDCA and a placebo [14]. Moreover, we reported that combination therapy was able to improve both the clinical score and long-term prognosis of PBC, especially at the early stage [15,16]. Regarding other fibrates, combined fenofibrate and UDCA could achieve significant biochemical improvements in patients with PBC and an incomplete response to UDCA [33,34] and reduced mortality, hepatic decompensation, and the need for liver transplantation [35]. However, fenofibrate therapy is contraindicated for patients with liver dysfunction in Japan. Although pemafibrate has fewer side effects, further direct comparative studies are needed to determine whether its therapeutic efficacy is superior.

Lastly, the combination of UDCA and fibrates in PBC patients might have other potential benefits, including improvements in such symptoms as itching and fatigue [35,36]. However, adverse reactions that include increased serum CK levels, decreased eGFR, and myalgia have been reported in bezafibrate or fenofibrate treatment [37]. An earlier study from Japan did not mention the adverse reactions of bezafibrate add-on as a study limitation [15]. In contrast, a very recent report described that pemafibrate addition to UDCA did not impact creatinine or eGFR [22], which corroborated our findings from over four years of observation. The drug also produced a lower incidence of adverse effects than did fenofibrate in patients with dyslipidemia [17,38]. Longer studies are warranted to clarify the clinical advantage of pemafibrate with UDCA, especially with regard to safety.

The present study had several limitations. It was retrospective in nature and contained a small sample size. Since it included PBC patients under long-term follow-up, some individuals had progressed to liver cirrhosis or HCC by the third or fourth year of observation. The discontinuation of pemafibrate was, therefore, unavoidable in some cases, resulting in missing laboratory data. Further investigation is needed regarding the long-term continuation of pemafibrate in the aging PBC population. Future studies should also evaluate the long-term prognosis of the entire cohort, including these patients. Since pemafibrate is not approved for national health insurance coverage as a treatment for PBC alone, its use in this study was limited to PBC patients with concomitant dyslipidemia. Therefore, the efficacy of pemafibrate in UDCA-refractory PBC patients without dyslipidemia remains unclear. Additional prospective longitudinal studies are needed to confirm any advantage of pemafibrate for PBC in the clinical setting.

## Conclusions

The addition of pemafibrate to UDCA therapy in PBC patients with dyslipidemia led to significant medium-term improvements in ALP, GGT, and ALT levels, with a sustained increase in the ALP normalization rate during four years of treatment. While pemafibrate showed a favorable safety profile without severe adverse effects, further prospective studies are needed to confirm its long-term clinical benefits, particularly in UDCA-refractory PBC patients.
